# Sandwich-Type DNA Micro-Optode Based on Gold–Latex Spheres Label for Reflectance Dengue Virus Detection

**DOI:** 10.3390/s20071820

**Published:** 2020-03-25

**Authors:** Jeningsih, Ling Ling Tan, Alizar Ulianas, Lee Yook Heng, Nur-Fadhilah Mazlan, Nur Diyana Jamaluddin, Nurul Yuziana Mohd. Yusof, Bahariah Khalid, Goh Choo Ta

**Affiliations:** 1 Southeast Asia Disaster Prevention Research Initiative (SEADPRI-UKM), Institute for Environment and Development (LESTARI), Universiti Kebangsaan Malaysia, Bangi 43600, Malaysia; jeningsih22@yahoo.co.id (J.); leeyookheng@yahoo.co.uk (L.Y.H.); fadysagiprincess89@gmail.com (N.-F.M.); diyanajamaluddin@gmail.com (N.D.J.); gohchoota@ukm.edu.my (G.C.T.); 2 Department of Chemistry, Faculty of Mathematics and Science, Universitas Negeri Padang, Padang 25131, Indonesia; alizar_chem@yahoo.co.id; 3 Department of Chemical Sciences, Faculty of Science and Technology, Universiti Kebangsaan Malaysia, Bangi 43600, Malaysia; 4 Department of Earth Sciences and Environment, Faculty of Science and Technology, Universiti Kebangsaan Malaysia, Bangi 43600, Malaysia; yuziana@ukm.edu.my; 5 Department of Medicine, Faculty of Medicine and Health Sciences, Universiti Putra Malaysia, Serdang 43400, Malaysia; bahariah@upm.edu.my; 6 Hospital Serdang, Jalan Puchong, Kajang 43000, Malaysia

**Keywords:** gold nanoparticles, latex particles, optical biosensor, reflectance, sandwich hybridization

## Abstract

A DNA micro-optode for dengue virus detection was developed based on the sandwich hybridization strategy of DNAs on succinimide-functionalized poly(*n*-butyl acrylate) (poly(*n*BA-NAS)) microspheres. Gold nanoparticles (AuNPs) with an average diameter of ~20 nm were synthesized using a centrifugation-based method and adsorbed on the submicrometer-sized polyelectrolyte-coated poly(styrene-*co*-acrylic acid) (PSA) latex particles via an electrostatic method. The AuNP–latex spheres were attached to the thiolated reporter probe (rDNA) by Au–thiol binding to functionalize as an optical gold–latex–rDNA label. The one-step sandwich hybridization recognition involved a pair of a DNA probe, i.e., capture probe (pDNA), and AuNP–PSA reporter label that flanked the target DNA (complementary DNA (cDNA)). The concentration of dengue virus cDNA was optically transduced by immobilized AuNP–PSA–rDNA conjugates as the DNA micro-optode exhibited a violet hue upon the DNA sandwich hybridization reaction, which could be monitored by a fiber-optic reflectance spectrophotometer at 637 nm. The optical genosensor showed a linear reflectance response over a wide cDNA concentration range from 1.0 × 10^−21^ M to 1.0 × 10^−12^ M cDNA (*R*^2^ = 0.9807) with a limit of detection (LOD) of 1 × 10^−29^ M. The DNA biosensor was reusable for three consecutive applications after regeneration with mild sodium hydroxide. The sandwich-type optical biosensor was well validated with a molecular reverse transcription polymerase chain reaction (RT-PCR) technique for screening of dengue virus in clinical samples, e.g., serum, urine, and saliva from dengue virus-infected patients under informed consent.

## 1. Introduction

Dengue virus is regarded globally as the most important arboviral disease. Dengue virus reproduces in temperate climate zones, e.g., in tropic and sub-tropic regions. The high environmental temperature increases the lifespan of female *Aedes aegypti* mosquitoes and shortens the extrinsic incubation period of dengue virus before transmitted to humans [1]. World Health Organization (WHO 2014) [2] reports that the heaviest burden of dengue fever is in the Asia Pacific countries. There are almost two billion people at risk of arbovirus diseases, and the increasing number of severe dengue cases overwhelms health facilities.

Conventional dengue virus detection methods include virus isolation in cell culture, amplification of RNA with reverse transcription polymerase chain reaction, and serological testing for detection of antigen and antibody [1]. However, traditional laboratory techniques are laborious and time-consuming, especially for the cultivation of dengue viruses, and the confirmation of dengue infection in human normally takes several days up to two weeks, whereas the sample collection can only be done after five days of illness onset. Hence, they are not able to provide real-time results. Due to the advent of technology, immunoglobulin G (IgG)-based ELISA assays and many rapid diagnostic test kits were developed, which are used for large-scale routine testing. Nevertheless, they show flavivirus cross-reactivity, while they are less sensitive and not type-specific [3]. As such, miniaturized, portable and user-friendly detection kits are highly required for high-sensitivity and high-specificity diagnosis of populations sustaining mosquito-borne viral disease.

In view of the great demand for high-sensitivity detectors and single-use sensors, field-effect transistor (FET)-based dengue virus sensors were developed for point-of-care measurements by virtue of their low cost and ultra-sensitivity in signal transduction. Furthermore, there are several other electrochemical-based biosensors reported for the determination of dengue virus. Cheng et al. [4] constructed an electrochemical biosensor based on a nanoporous alumina membrane for quantitative determination of dengue virus particles. Nonetheless, this biosensor suffered from a non-specific virus particle interference effect. Zhang et al. [5] fabricated a silicon nanowire-based FET DNA biosensor for dengue virus detection. Nevertheless, the preparation of silicon nanowire involved tedious physical etching and a high-temperature oxidation step followed by lithographic treatment. Additionally, a highly sensitive impedimetric DNA biosensor was developed on the gold nanoparticle–polyaniline composite-modified gold electrode by Nascimento et al. [6] for picomolar detection of the dengue serotype. Furthermore, optical biosensors also attracted much attention owing to their advantages of simplicity and high sensitivity. Fletcher et al. [7] designed an optical modular biosensor for dengue virus DNA detection. The biosensor was made of a restriction endonuclease-modified aptamer, an oligonucleotide linker module, and a fluorescent label. Nevertheless, modification of the aptamer with an enzyme could potentially affect the aptamer–target binding, because DNA aptamer–target binding requires pre-folding of the single-stranded oligonucleotide into a specific tertiary structure prior to binding with its target molecule via van der Waals forces, hydrogen bonding, and electrostatic interaction in order to allow this three-dimensional structure aptamer to function like an antibody. Chemical modification of the aptamer with an enzyme would result in the binding site becoming inactivated due to the conformational change in the aptamer, with the denatured aptamer failing to bind with its target molecule.

Detection of dengue virus DNA using a similar reflectometer transducer was reported by Baeumner and co-workers [8] with different DNA sensor design using a streptavidin-modified polyethersulfone membrane as a DNA supporter with a dye-entrapping liposomal nanovesicle rDNA label. However, the linear range obtained was found to be fairly narrow, which could be attributed to the two-dimensional thin membrane being used as the immobilization matrix, which restricted loading of the DNA probe. The currently available technologies for dengue virus detection appear to be fairly complex and time-consuming. Therefore, there is still a need to develop a simple and friendly operating system to detect dengue virus.

**Figure 1:**
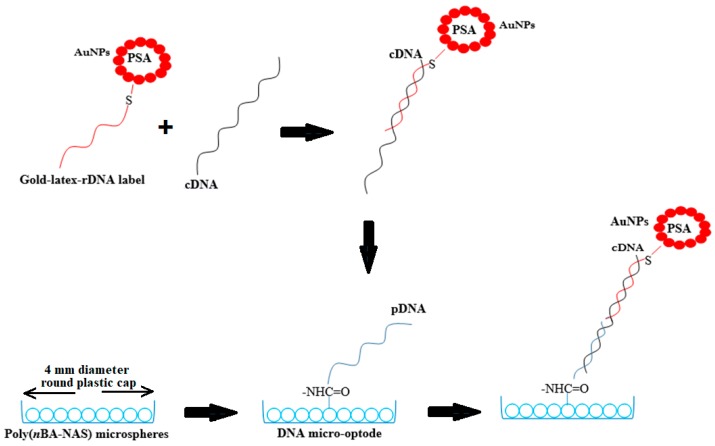
Schematic diagram showing the one-step sandwich hybridization recognition procedure, which involves a DNA capture probe (pDNA) immobilized on the acrylic microspheres, and target (cDNA) and reporter (rDNA) probes labeled with gold nanoparticle–poly(styrene-*co*-acrylic acid) latex (AuNP–PSA) spheres.

Herein, we propose a facile DNA micro-optode for the optical reflectance determination of dengue virus based on a DNA sandwich detection strategy. A DNA micro-optode is defined herein as an optical sensor based on a DNA recognition probe and submicrometer-sized gold nanoparticle (AuNP)-labeled polyelectrolyte-coated poly(styrene-*co*-acrylic acid) (PSA) latex particles as the optical label and poly(*n*-butyl acrylate) (poly(*n*BA-NAS)) microspheres as the DNA hybridization platform. Latex microspheres of PSA were synthesized with a one-pot reaction in the aqueous phase and modified layer-by-layer with polyelectrolytes to form multilayer PSA latex particles before being electrostatically attached with AuNPs and chemically grafted to the signal probe (rDNA) by thiol chemistry to produce a gold–latex rDNA label specific to dengue virus serotype 2 nucleic acid sequences. Poly(*n*BA-NAS) microspheres, i.e., the copolymer bio-carrier matrix, on the other hand, were synthesized via a miniemulsion photo-polymerization technique and conjugated to the aminated DNA capture probe (pDNA) via a peptide covalent bond. The spherical morphology of the acrylic microspheres would confer greater surface area in the three-dimensional structure for higher loading of the recognition probe for high-sensitivity detection of target DNA. The DNA sandwich detection procedure was carried out by pre-hybridizing the AuNP–PSA optical amplify reporter label with target DNA (cDNA) prior to hybridization with immobilized pDNA, thereby bringing violet coloration to the acrylic microsphere DNA supporting matrix. The color change of the DNA biosensor was visualized using a fiber-optical reflectance spectrophotometer for quantitation of dengue virus cDNA concentration. The proposed sandwich-type optical DNA biosensor could be simply transformed into a portable colorimetric sensor for naked-eye in situ dengue virus detection. Figure 1 depicts the DNA hybridization procedure for the proposed sandwich-type optical DNA biosensor toward dengue virus detection. 

## 2. Materials and Methods

### 2.1. Reagents and Materials

All the chemicals were of analytical grade and were used without any further purification. *n*-Butyl acrylate (nBA, 99%), 2-2-dimethoxy-2-phenylacetophenone (DMPP, 99%), 1,6-hexanediol diacrylate (HDDA, 90%), acrylic acid (99%), poly(allylamine) hydrochloride (PAA, molecular weight (Mw) 50,000), and poly(sodium 4-styrenesulfonate) (PSS, Mw 70,000) were obtained from Aldrich. Sodium dodecyl sulfate (SDS, 99%) and trisodium citrate dihydrate (C_6_H_5_Na_3_O_7_∙2H_2_O, 99%) were supplied by Systerm. *N*-Acryloxysuccinimide (NAS, 99%) and sodium chloride (NaCl, 99%) were purchased from Across. Gold(III) chloride trihydrate (HAuCl_4_∙3H_2_O, ≥49% Au basis) and styrene (99%) were procured from Sigma-Aldrich, and ammonium persulfate (APS, ≥98%) was acquired from Sigma. All the oligonucleotides used in this study, e.g., a single-stranded amine-modified DNA capture probe (amino modifier positioned at the 5’-end of the 15-mer pDNA with a C6 spacer arm), thiolated reporter probe (dithiol inserted into a 15-mer rDNA at the 3’ position), target DNA (cDNA, 30-mer), one-base mismatched DNA (1mDNA, 30-mer), and non-complementary DNA (ncDNA, 30-mer) were synthesized by Sigma-Aldrich, and are tabulated in Table 1. DNAs from supplier stock at 100 µM were diluted with 0.1 M potassium phosphate buffer (pH 7.0) to obtain a 1 µM DNA stock solution each. Potassium phosphate (K-phosphate) buffer at 0.05 M and pH 7.0 was prepared by appropriately mixing 0.05 M dipotassium hydrogen phosphate (K_2_HPO_4_, >99%, Fluka) with 0.05 M potassium dihydrogen phosphate (KH_2_PO_4_, 99.5%, Fluka). All the aqueous solutions were prepared using Milli-Q water.

### 2.2. Instrumentation

Ultraviolet (UV)-initiated photo-polymerization of poly(*n*-butyl acrylate-*co*-*N*-acryloxysuccinimide) (poly(*n*BA-NAS)) microspheres was performed using a UV-exposure unit (RS Components 196–5251) emitting UV light at the wavelength of 350 nm. The size and morphology of both acrylic and latex microspheres were examined with a field-emission scanning electron microscope (FESEM, Zeiss Merlin/Merlin Compact/Supra 55VP). Attenuated total reflectance (ATR) results were acquired from an Agilent Cary 630 FTIR spectrometer. The average size of AuNPs was characterized by transmission electron microscopy (TEM, Thermo Scientific Talos L120C) and UV–Vis spectrophotometry (Varian-Cary Win UV 50). DNA biosensor optimization studies were carried out using an Ocean Optics USB4000 Spectrometer coupled to a bifurcated optical fiber with a DH-2000-BAL UV–Vis—near-infrared (NIR) light source in the wavelength range of 200–1099 nm.

### 2.3. Synthesis of Poly(*n*-butyl acrylate-*co*-*N*-acryloxysuccinimide) Microspheres

Succinimide-functionalized acrylic microspheres were synthesized based on the method reported by Ulianas et al. [9] with some modifications. The mixture containing 7 mL of *n*BA monomers, 0.01 g of SDS surfactant, 450 μL of HDDA cross-linker, 0.1 g of DMPP photo-initiator, 6 mg of NAS monomers, and 15 mL of Milli-Q water was sonicated for 10 min. The resulting emulsion suspension was photo-cured for 600 s under UV radiation with a continuous nitrogen (N_2_) gas flow. The as-synthesized succinimide-modified polyacrylate microspheres were then collected by centrifugation at 4000 rpm for 30 min and washed with abundant 0.05 M K-phosphate buffer at pH 7.0 followed by air drying at ambient temperature (25 °C).

### 2.4. Preparation of Gold–Latex Particles

Gold nanoparticles (AuNPs) were synthesized by a centrifugation-based method according to Liang et al.’s [10] recommended protocol. In brief, 100 mL of 0.01% (*w*/*v*) HAuCl_4_∙3H_2_O was firstly heated at 90 °C with vigorous stirring. Then, 3.5 mL of 1% (*w*/*v*) C_6_H_5_Na_3_O_7_∙2H_2_O was added, and the solution was stirred for another 15 min. The solution turned into a faint blue color after 25 s of reaction. A burgundy red colloidal solution was formed after approximately 30 min of reaction, indicating the formation of AuNPs. The resulting gold colloid solution was then left to cool to room temperature before it was centrifuged for 20 min at 4 °C with a centrifugal force (RCF) of 11,930× *g*. Poly(styrene-*co*-acrylic acid) (PSA), i.e., the latex microspheres, on the other hand, was synthesized as described by Kuan et al. [11] with slight modifications. Firstly, 190 g of Milli-Q water was purged with N_2_ gas in a three-necked flask submerged in a water bath for 1 h under continuous stirring at 350 rpm. Then, 20 g of styrene and 0.5 g of acrylic acid were then added at 70 °C, followed by adding 10 mL of 0.02 g∙mL^−1^ APS into the reaction pot, which was allowed to react for another 7 h under vigorous stirring and nitrogen atmosphere. The resulting PSA copolymer microspheres were isolated by centrifuging twice with Milli-Q at 13,000 rpm for 20 min. Next, the latex microspheres were sequentially incubated in 1 mg∙mL^−1^ PAA, PSS, and PAA polyelectrolytes containing 0.5 M NaCl. Each polyelectrolyte incubation step was performed for 30 min, followed by washing with a plentiful amount of Milli-Q water. The as-prepared multilayer PSA microparticles were stored in Milli-Q water when not in use. After that, AuNPs were electrostatically adsorbed onto the polyelectrolyte-coated latex spheres by mixing multilayer PSA particles with colloidal AuNPs in the volume ratio of 1:20 and incubating for 30 min. The gold–latex particles were finally recovered by filtration using a 0.2-μm cellulose acetate filter, and then stored in 0.5 mL of Milli-Q water in a refrigerator at 4 °C until use. 

### 2.5. Fabrication of Sandwich-Type DNA Micro-Optode

The sandwich-type optical DNA biosensor was constructed by depositing the 0.12 mg∙µL^−1^ succinimide-modified acrylic microsphere suspension in ethanol in a round plastic cap of diameter 4 mm. Then, 30 µL of 4 μM pDNA was deposited on the acrylic microsphere surface and kept in ambient conditions for 24 h for covalent immobilization reaction to take place. Subsequently, the DNA biosensor was rinsed with 0.05 M K-phosphate buffer (pH 7.0) to remove the unbound DNA probes from the polyacrylate microspheres. In a separate 200-µL microcentrifuge reaction tube, 10 µL of colloidal gold–latex particles in Milli-Q water were reacted with 10 µL of 20 nM rDNA for 1 h at 25 °C. Next, 10 µL of 1 × 10^−12^ M (1 pM) cDNA was added to the AuNP–latex–rDNA complex, and DNA hybridization was allowed to occur for another 1 h before application on the DNA biosensor microsurface for hybridization with immobilized pDNA. The DNA micro-optode was thoroughly washed with copious amounts of 0.05 M K-phosphate buffer at pH 7.0 prior to optical reflectance measurement by using a fiber-optic reflectance spectrophotometer.

### 2.6. Optimizing the DNA Micro-Optode for Dengue Virus Detection

In order to determine the linear response range of the DNA biosensor, the cDNA concentration was varied between 1.0 × 10^−21^ M (zM) and 0.1 × 10^−3^ M (0.1 mM), whilst the immobilized pDNA and rDNA were maintained at 4 μM and 20 nM, respectively. A selectivity study was carried out by comparing the reflectance intensity of the DNA biosensor toward dengue virus cDNA, ncDNA, and 1mDNA at 1 × 10^−16^ M and 637 nm. A buffer pH effect study was conducted by changing pH of the DNA hybridization medium between pH 4.4 and pH 8.9 using 0.05 M K-phosphate buffer toward optical reflectance detection of 1 pM dengue virus cDNA at 637 nm. To evaluate the response time of the sandwich-type DNA biosensor, the DNA biosensor was hybridized with 1 pM cDNA in the presence of the gold–latex reporter label, and the reflectance response at 637 nm was taken every 30 min for 360 min. A regeneration test was performed by repeating the oligonucleotide sandwich hybridization reaction of the DNA biosensor with 1 pM cDNA labeled with gold–latex–rDNA conjugates after incubation in a 0.1 M NaOH regeneration solution for 10 min at 25 °C until an appreciable decrement of the optical response was registered at 637 nm. In order to determine the shelf life of the proposed optical DNA biosensor based on the gold–latex sphere label, 21 units of acrylic microsphere-based DNA biosensors were batch-produced and stored in ambient conditions. The optical reflectance response of the three units of DNA micro-optodes were determined by applying the DNA biosensors for dengue virus detection at 1 pM on a daily basis until a significant decline in the reflectometric response was observed at 637 nm.

### 2.7. Validation Study of DNA Micro-Optode with RT-PCR for Dengue Virus Detection in Blood, Urine, and Saliva Samples

A total of three patients, i.e., a young Malaysian Malay man, a middle-aged Malaysian Chinese woman, and a middle-aged Malaysian Malay man were identified with dengue virus infection, and, along with a healthy young Malay women volunteer, they participated in this study with an informed consent form signed accordingly. Human participants’ personal information is being kept confidential and the privacy is being respected following the Personal Data Protection Act 2010 (PDPA-Act 709). Firstly, 6 mL of a blood sample was collected into plain red-top VACUTAINER^®^ tubes without anticoagulant. Urine and saliva samples were collected into the respective plastic containers. All the samples were immediately supplemented with ethylenediaminetetraacetic acid (EDTA) to a final concentration of 40 mM in 1.5-mL microcentrifuge tubes upon arrival at the Molecular Biology Lab of Universiti Kebangsaan Malaysia and flash-frozen in liquid nitrogen prior to storage at −80 °C. The study was conducted in accordance with the Declaration of Helsinki, and the protocol was approved by the National University of Malaysia Research Ethics Committee (UKMREC) [JEP-2017-406] and the Ministry of Health Medical Research and Ethics Committee (MREC) [NMRR-17-1574-36144]. 

The dengue virus genome is a single strand of RNA. It is referred to as positive-sense RNA because it can be directly translated into proteins. However, the RNA structure is inherently weaker than DNA as RNA is made up of ribose units, which have a highly reactive hydroxyl group on C2 that takes part in RNA-mediated enzymatic events. This makes RNA more chemically labile than DNA. Furthermore, RNA is also more prone to heat degradation compared to DNA. As such, RNAs were extracted from biological fluids collected from dengue virus-infected patients and a healthy volunteer in this study, and they were converted into their complementary DNA (cDNA) sequences by reverse transcriptase, followed by amplification of the newly synthesized cDNA by standard PCR procedures before the optical biosensor measurement was made. 

In detail, 200 µL of the respective blood (B), urine (U), and saliva (S) samples collected from patient 1 (P1), patient 2 (P2), patient 3 (P3), and the healthy volunteer (H) were centrifuged at 3000 rpm for 10 min prior to RNA extraction using the QIAamp Viral RNA Mini Kit (QIAGEN, Hilden, Germany) according to the manufacturer’s protocol. The isolated RNA was then eluted using TE buffer (10 mM Tris-CL and 1 mM EDTA) as the elution buffer to a final volume of 60 µL, and the concentration of extracted RNA was determined by using a NanoDrop spctrophotometer. The synthesis of cDNA from RNA was done via reverse transcription polymerase chain reaction (RT-PCR) by using a ReverTra Ace qPCR RT Master Mix Kit (Toyobo, Japan). Then, 100 ng of the isolated RNA was used as the RNA template in the presence of 4 μL of RT mix and topped up with nuclease-free water to a final volume of 20 μL. The reaction mixture was incubated at 37 °C for 15 min before heating at 98 °C for 5 min. The resulting cDNA obtained was employed in the subsequent PCR amplification step as the cDNA template (2 μL) using a conventional PCR Top Taq Master Mix (QIAGEN, Hilden, Germany) with a dengue virus serotype 2-specific forward primer (5′–CATGGCCCTGGTGGCG–3′) and reverse primer (3′–CCCCATCTCTTCAATATCCCTG–5′) at 300 nM, 6.25 μL of Top Taq DNA polymerase, 1.65 μL of nuclease-free water, and 2 μL of CoralLoad Gel loading dye in a total reaction volume of 12.5 μL. PCR amplification was performed with an initial denaturation at 94 °C for 3 min followed by 35 cycles of denaturation at 95 °C for 45 s, annealing at 56 °C for 30 s, extension at 72 °C for 1 min, and final extension step at 72 °C for 5 min. The PCR product was confirmed by running gel electrophoresis at 90 V for 60 min on 4% agarose gel stained with RedSafe Nucleic Acid Staining Solution in 1× TBE buffer (Tris/borate/EDTA) [5,12].

With respect to dengue virus DNA detection in blood, urine, and saliva samples with the optical DNA biosensor, 10 µL of AuNP–latex microspheres in Milli-Q water were firstly reacted with 10 µL of 20 nM rDNA for 1 h at room temperature. Then, 10 µL of the resulting cDNA acquired from the cDNA synthesis step was then introduced into the gold–latex–rDNA label and left for 1 h before the total reaction mixture at 30 µL was deposited onto the DNA biosensor surface. After 90 min of DNA hybridization duration, the DNA biosensor was rinsed with abundant 0.05 M K-phosphate buffer (pH 7.0) before the reflectance measurement was recorded at the working wavelength of 637 nm. The results obtained by both the reflectance DNA biosensor and the standard RT-PCR method were statistically compared using a paired *t*-test at four degrees of freedom and a 95% confidence level (*p* = 0.05) [8].

## 3. Results and Discussion

### 3.1. Characterization of Gold Nanoparticles, Polyacrylate, and Latex Microspheres 

The FESEM image in Figure S1a (Supplementary Materials) shows the spherical morphology of the as-prepared poly(*n*BA-NAS) microparticles with diameter in the range of 0.5–3.0 µm. The rather large size distribution of the spheres indicates that non-uniformity of the acrylic microspheres was obtained. The spherical morphology of polyacrylate is essential in the immobilization process as it maximizes the surface area available for subsequent pDNA immobilization via covalent binding. The FESEM micrograph of PSA colloidal particles in Figure S1b presents a rather homogenous size distribution between 167 nm and 216 nm (see Supplementary Materials). Polyacrylate microspheres modified with NAS succinimide ester groups were chosen as a substrate carrier as they allowed strong covalent binding with aminated pDNA via a succinimide–amine coupling reaction. In addition, the excellent adhesive characteristic of the poly(*n*BA-NAS) microspheres allowed them to be stably immobilized in the plastic cap during the DNA biosensor fabrication process. PSA microspheres, on the other hand, given their far smaller size, i.e., about 14 to 18 orders of magnitude compared to acrylic microspheres, provided a large specific surface area for the high loading capacity of the AuNP optical amplify reporter label through electrostatic interaction in order to generate the substantial optical amplification effect of the reflectance response upon sandwich DNA detection of targeted DNA sequences.

The PSA microspheres possessed carboxylic acid moieties at the particle surface that enabled further modification of the latex particles with polyelectrolyte via an electrostatic method, subsequently harnessing their electrostatic interactions with citrate-reduced AuNPs. The small latex particle size indicates the high specific surface area available for the high loading capacity of the AuNP optical amplify reporter label through electrostatic interaction. The FESEM image of PSA microparticles decorated with AuNPs is shown in Figure S1c (Supplementary Materials).

The FTIR spectra of succinimide-modified acrylic microspheres and PSA latex microparticles are demonstrated in Figure S1d (Supplementary Materials). The C–O stretching vibrations of *n*BA and the succinimide ester of NAS moieties of poly(*n*BA-NAS) can be perceived in the wavenumber range of 1114.48 cm^−1^ to 1064.46 cm^−1^. The strong and sharp C=O absorption band of *n*BA and NAS moieties can be observed at 1732.29 cm^−1^. The adsorption bands at 1446.21 cm^−1^, 1376.32 cm^−1^, and 2958.58–2870.06 cm^−1^ correspond to the –CH_2_ bending, –CH_3_ bending, and *sp*^3^ C–H stretching vibrations of succinimide-functionalized acrylic microspheres. The presence of C–N stretching frequency in the wavenumber range of 1236.55 cm^−1^ to 1153.62 cm^−1^ indicates that the polyacrylate microspheres were successfully modified with NAS succinimide ester groups to allow covalent binding with aminated pDNA via a succinimide–amine coupling reaction [13].

PSA latex microspheres were synthesized by styrene and acrylic acid monomers, with their FTIR spectrum presenting several characteristic peaks of both styrene and acrylic acid. The carbonyl and hydroxyl functional groups in the acrylic acid segment of the latex copolymer was represented by the absorption bands at 1730.41 cm^−1^ and 2911.06 cm^−1^, respectively. The absorption peak at 1605.55 cm^−1^ was ascribed to the carbon–carbon stretching vibrations in the aromatic ring of the styrene segment of the copolymer. Furthermore, *sp*^3^ C–H stretching can be seen at 3002.81 cm^−1^, whilst the methylene (–CH_2_) group had a characteristic bending absorption of 1451.79 cm^−1^. Polymerization between vinyl functional groups of styrene and acrylic acid to form a C–C covalent bond can be perceived at the wavenumber of 1493.74 cm^−1^.

The UV–Vis spectrum of colloid AuNPs synthesized via a centrifugation-based method exhibited a characteristic plasmon absorption peak at 520 nm (Figure S1e, Supplementary Materials), indicating that the as-synthesized colloidal AuNPs had diameters of ≤30 nm [14]. Nevertheless, particle size determination using an advanced TEM technique revealed that the measured average diameter of AuNPs was 20 ± 5 nm (Figure S1f, Supplementary Materials).

### 3.2. Characterization of DNA Micro-Optode with Fiber-Optic Reflectance Spectrophotometer

**Figure 2:**
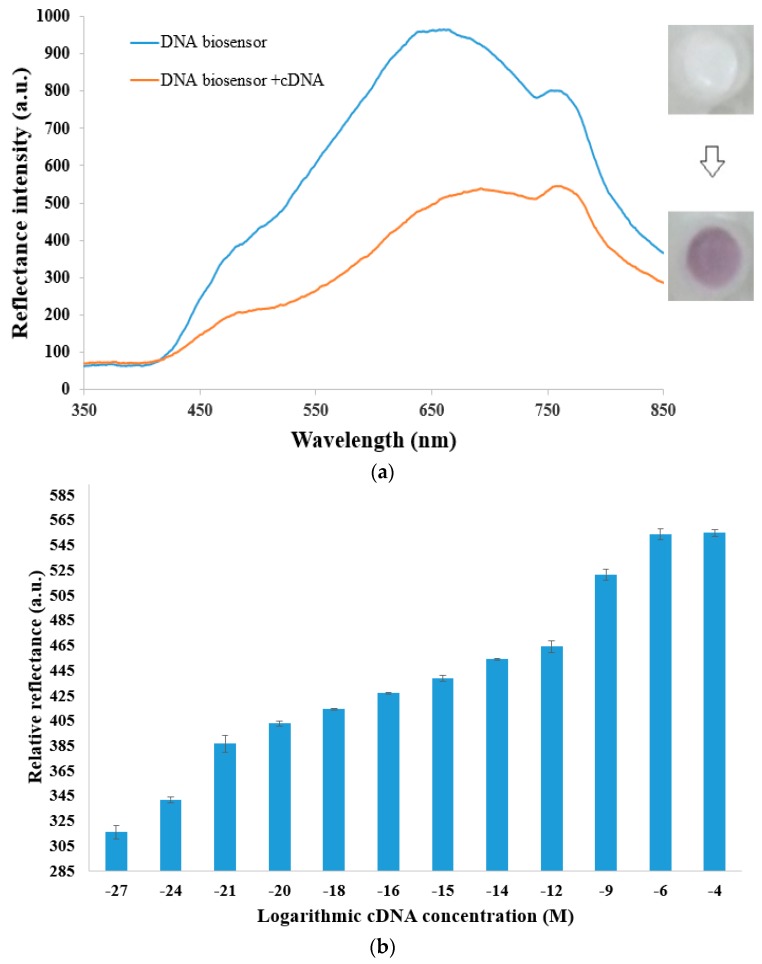

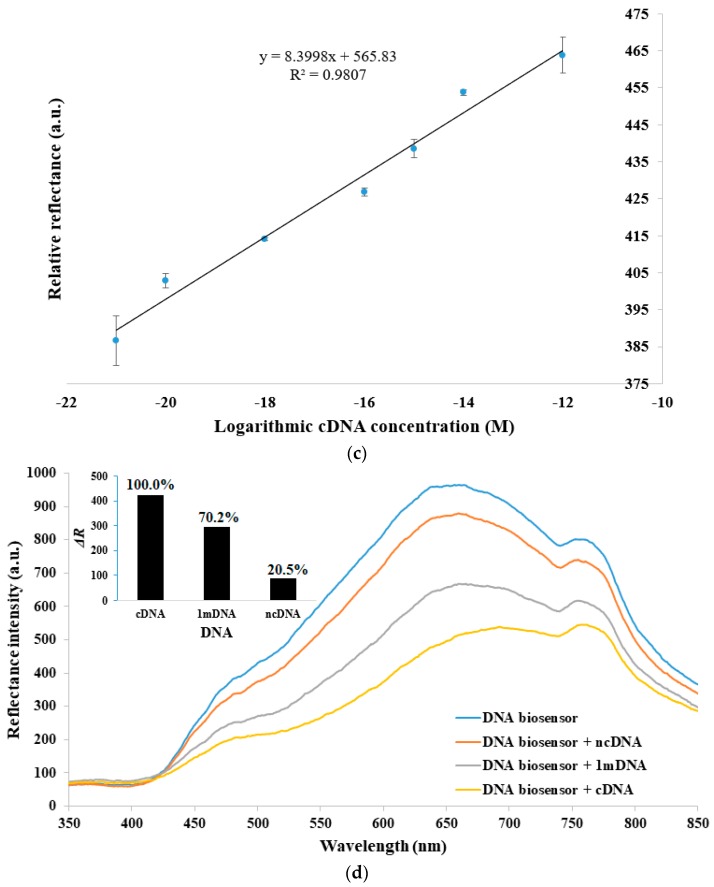
(**a**) Optical reflectance detection of 1 pM dengue virus cDNA at pH 7.0 and 637 nm via sandwich hybridization process using 4 µM immobilized pDNA and 20 nM immobilized rDNA with the gold–latex sphere label. The inset shows the color appearance of the DNA micro-optode before and after reaction with the target DNA–AuNP–latex. (**b**) Relative reflectance response at 637 nm between DNA biosensor reflectance spectra before and after sandwich hybridization reaction in the cDNA concentration range of 1 × 10^−27^ M to 1 × 10^−4^ M (*n* = 3). (**c**) The linear response range of the DNA biosensor between 1.0 zM and 1.0 pM (*n* = 3). (**d**) Selectivity assessment of the DNA biosensor toward the determination of cDNA, ncDNA, and 1mDNA at 1 × 10^−16^ M and 637 nm.

The DNA biosensor, which refers to the pDNA immobilized acrylic microspheres, presented a cloudy deep white color that gave maximum reflectance response at the wavelength of 637 nm. Pre-hybridization of cDNA to the gold–latex reporter label was performed before application on the DNA biosensor. A distinctive violet coloration was developed on the DNA biosensor surface as sandwich hybridization of the cDNA–rDNA–AuNP–PSA complex to the immobilized pDNA was performed (i.e., DNA biosensor + cDNA). This was attributed to the aggregation of AuNP-labeled multilayer latex on the DNA biosensor micro-surface, resulting in a substantial decline in the optical reflectance response at 637 nm. The formation of a violet-hued cDNA–rDNA–AuNP–PSA complex on the DNA biosensor absorbed incident light from the feed fiber of the bifurcated optical fiber and attenuated the reflectance signal over the visible wavelength. This was in contrast to the white-colored background of the DNA biosensor, which gave higher reflectance intensity, as a bright color (white) reflects better than a dark one (violet). The submicrometer-sized polyelectrolyte-coated latex particles, which ended with a strong cationic PAA polyelectrolyte layer that possessed a number of positive charges along its backbone chain, facilitated electrostatic binding with negatively charged AuNPs at the latex microsphere surface. The DNA biosensor optical reflectance response before and after the oligonucleotide sandwich hybridization reaction for detection of dengue virus cDNA is demonstrated in Figure 2a. The difference between the reflectance value (relative reflectance, ΔR) of the DNA biosensor, i.e., pDNA immobilized acrylic microspheres at 637 nm, and that of the sandwich hybridization of the cDNA–rDNA–AuNP–PSA complex to the immobilized pDNA at the same wavelength was adopted as the measurement signal, i.e., ΔR = RpDNA − RcDNA. In view of the greatest ΔR, i.e., an enormous divergence between the DNA biosensor reflectance response (RpDNA) and DNA biosensor + cDNA reflectance signal (RcDNA), occurring at 637 nm, this wavelength was employed as the working wavelength in the subsequent experiments.

The increasing relative reflectance signal at 637 nm between the DNA biosensor reflectance spectra before and after the DNA sandwich hybridization process upon increasing the cDNA concentration from 1 × 10^−27^ M to 1 × 10^−4^ M is illustrated in Figure 2b. At low cDNA concentration, i.e., below 1 zM, the DNA biosensor showed an almost flat reflectance signal. By increasing the dengue virus cDNA concentration from 1 zM to 1 pM, the reflectance response of the acrylic microsphere-based DNA biosensor decreased proportionally as the amount of AuNP-decorated PSA spheres agglomerated on the DNA biosensor platform increased with increasing target DNA concentration, whereby a proportional ΔR enhancement of the DNA biosensor response from 1 zM to 1 pM was noted. The reflectance DNA biosensor response increased abruptly from 1 pM to 1 µM as there was a further increasing number of AuNP-modified latex spheres aggregated on the DNA biosensor, and the DNA biosensor developed from a white to a distinctive violet hue. A steady-state reflectance response was attained as the cDNA concentration increased above 1.0 µM due to an optimum sandwich DNA hybridization reaction being achieved at the DNA biosensor surface, where a further increase in the targeted DNA concentration would not result in any increment of relative reflectance response at 637 nm. The dynamic linear concentration range of the DNA micro-optode based on the gold–latex sphere label, which was estimated using an Excel trend function, was determined to be in the range of 1.0 zM to 1.0 pM cDNA on a logarithmic scale with a linear correlation coefficient value (*R^2^*) obtained at *R^2^* = 0.9807 (Figure 2c). The limit of detection (LOD) of the reflectometric sandwich-type DNA micro-optode, which was determined based on the average of the blank reflectance DNA biosensor response plus three standard deviations, was estimated at 1 × 10^−29^ M cDNA. For each cDNA assay, three trials were run using DNA a micro-optode prepared from several batches, and a satisfying reproducibility relative standard deviation (RSD) in the range of 0.1%–1.7% (*n* = 3) of ΔR values was estimated, showing that high reproducibility of the sandwich-type optical reflectance DNA biosensor performance was still achievable regardless of the non-uniform polyacrylate microsphere size. 

The selectivity study of the DNA biosensor toward the detection of dengue virus cDNA is presented in Figure 2d. Both immobilized pDNA and rDNA labeled with gold–latex spheres appeared to be highly specific toward dengue virus cDNA, and they were able to distinguish oligonucleotides with a single base mutation. The hybridization reaction was effective only with cDNA with a maximum ΔR response attained at 637 nm. The ΔR value of the sandwich-type optical DNA biosensor with ncDNA showed some 20.5% at 637 nm as the ncDNA strand contained 11 bases, which are complementary with both immobilized pDNA and rDNA. The 1mDNA gave 70.2% ΔR response with the reflectance DNA biosensor, implying that the proposed sandwich-type DNA biosensor is able to discriminate even a single mismatched base pair. As the DNA micro-optode was capable of identifying point mutations in DNA sequences, this signifies that the sandwich-type DNA micro-optode has excellent specificity to refrain from any false positive results that can be yielded due to antigenic cross-reactivity among different dengue virus serotypes and different flavivirus members. Non-specific adsorption of AuNPs onto the polyacrylate microsphere surface was negligible as the succinimide functional group with a negatively charged oxygen atom on the acrylic microspheres repelled like charges from the AuNP–PSA reporter label.

### 3.3. pH Effect, Response Time, Regeneration, and Long-Term Stability of the DNA Micro-Optode

**Figure 3:**
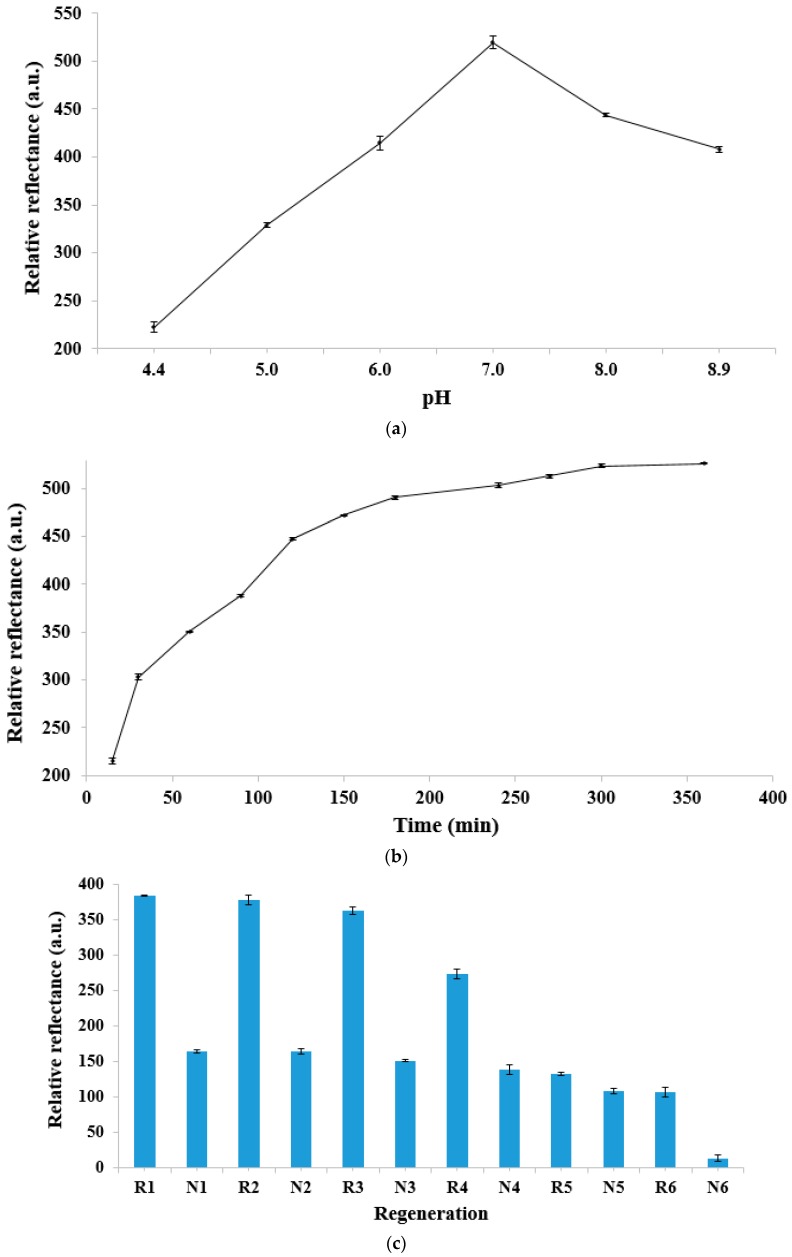

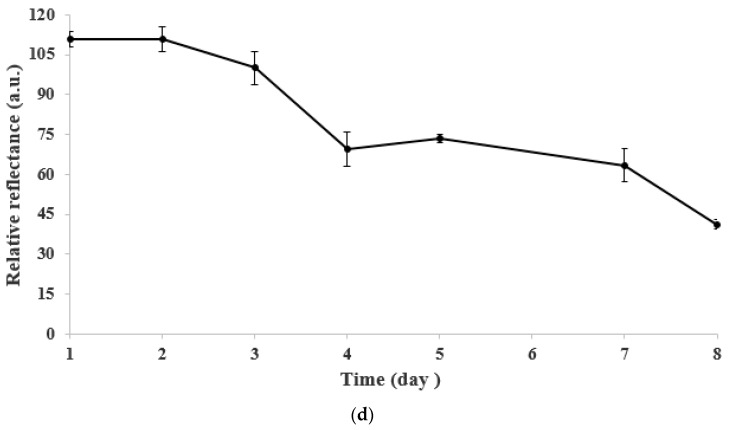
(**a**) The sandwich-type DNA micro-optode response toward the detection of 1 pM cDNA in 0.05 M K-phosphate buffer from pH 4.4 to pH 8.9 at 637 nm (*n* = 3). (**b**) The response time curve of the optical DNA biosensor upon sandwich DNA detection of 1 pM cDNA at 637 nm (*n* = 3). (**c**) Reversibility of the DNA biosensor after sandwich DNA hybridization treatment with 1 pM cDNA at pH 7.0 for 90 min (R) and regeneration with 0.1 M NaOH solution for 10 min (N) in ambient conditions (*n* = 3). (**d**) The long-term stability profile of the optical DNA biosensor for sensing of 1 pM dengue virus DNA for an experimental time period of seven days (*n* = 3).

The buffer pH effect toward sandwich DNA detection of dengue virus in 0.05 M K-phosphate buffer from pH 4.4 to pH 8.9 is depicted in Figure 3a. It was found that the relative optical reflectance signal at 637 nm decreased in both acidic and basic buffer solutions, and the maximum relative reflectance signal was obtained with 0.05 M K-phosphate buffer at neutral pH. In lower pH conditions, i.e., between pH 4.4 and pH 6.0, the acidic environment promoted a protonation reaction over the phosphodiester chain in the DNA sugar–phosphate backbone, thereby reducing aqueous solubility of the DNA molecule [15,16] and jeopardizing the sandwich DNA hybridization reaction between immobilized pDNA, cDNA, and the AuNP–PSA reporter label. At neutral pH, without a protonation reaction occurring at the DNA molecule phosphodiester chain, the water solubility of the DNA molecule was promoted, favoring the sandwich DNA hybridization reaction. At alkaline pH, i.e., from pH 8.0 to pH 8.9, the basic pH conditions broke up the hydrogen bonding between the nitrogenous base pairs of DNAs and denatured the double helical structure of DNAs [17]. Therefore, 0.05 M K-phosphate buffer pH was maintained at pH 7.0 throughout the sandwich DNA hybridization process in this study. 

Figure 3b exhibits the response time trend of the DNA micro-optode upon sandwich DNA detection of 1 pM cDNA at 637 nm. The relative reflectance intensity was noted to gradually increase with time from 30 min to 180 min as the number of gold–latex sphere labels accumulated on the DNA biosensor surface increased with increasing sandwich DNA hybridization reaction rate, thereby allowing a considerable optical amplification of the relative reflectance signal at 637 nm. The optical signal of the DNA micro-optode reached a plateau after 180 min of reaction time when the sandwich DNA hybridization reaction reached to an equilibrium state. Since 90 min of DNA hybridization reaction time gave a sufficient significant reflectance signal for optical quantification of the dengue virus DNA concentration, the sandwich DNA hybridization duration was fixed at 90 min prior to the reflectance measurement in the optimization of the fabricated optical genosensor. 

A reversibility study was conducted to determine the reusability of the optical DNA biosensor for assay of DNA after treatment with a mild NaOH regeneration solution. As can be seen in Figure 3c, the DNA biosensor was capable of giving a repeatable reflectance response for three consecutive DNA detections of 1 pM cDNA with a relative standard deviation (RSD) of 2.9%. A substantial decline in the relative reflectance signal of the DNA biosensor was observed starting after the fourth cycle of DNA analysis, which was attributed to the dissociation of hydrogen bonds between nucleobases of the DNA double-helix, as well as permanent desorption of the pDNA strand from the acrylic immobilization micro-matrix after repetitive regeneration of the DNA biosensor with 0.1 M NaOH solution [18]. As such, the sandwich-type DNA micro-optode based on the gold–latex sphere label can be reused for optical dengue virus detection up to a maximum of three times.

Based on the long-term stability profile of the sandwich-type reflectance DNA biosensor shown in Figure 3d, the DNA biosensor maintained its original high reflectance response toward sensing of 1 pM dengue virus DNA for the first two days of storage duration, and the DNA biosensor was found to be capable of retaining about 90% of its initial optical reflectance response on the third experimental day. The optical DNA biosensor response was then dropped to about 65% of its original response upon sandwich DNA detection of 1 pM dengue virus cDNA on the fourth storage day, followed by a further decline to about 55% of the DNA biosensor initial reflectometric response on the seventh day of storage time. Such a profound response decrement perceived on the seventh storage day of the DNA biosensor may be ascribed to the degradation of immobilized pDNA, which impeded the sandwich DNA hybridization process from taking place on the poly(*n*BA-NAS) microsphere surface. The immobilized pDNA had a serious degradation on the poly(*n*BA-NAS) microsphere on the seventh storage day as the DNA biosensors were stored in ambient conditions without proper preservation, and this may have denatured the immobilized pDNA as a result of exposure to heat, water, and sunlight, which can cause the DNA molecule to degrade faster. It can be deduced that the sandwich-type optical DNA biosensor possessed a two-day operational shelf life.

### 3.4. Validation of Sandwich-Type DNA Biosensor with RT-PCR for Dengue Virus Detection

The RNAs from blood (B), urine (U), and saliva (S) samples of three patients (i.e., P1, P2, and P3) suspected to be infected with dengue virus and of a healthy volunteer (H) were successfully isolated, and the concentrations of the respective RNAs determined by means of NanoDrop spectrophotometer at the wavelength of 260 nm and 280 nm are tabulated in Table S1 (Supplementary Materials). The concentrations of RNAs extracted from the respective human bodily fluids were obtained in the range of 4.9–60.6 ng/µL, whilst absorbance values in the A260/230 and A260/A280 ratios fluctuated between 0.02 and 2.60. Ideally, the absorption of nucleic acid samples between 1.9 and 2.0 in the absorbance ratio at 260/230 nm and 260/280 nm indicates that highly purified RNA was attained, and deviation from the ideal absorbance range is commonly due to the presence of molecular impurities in the isolated nucleic acid, e.g., protein, residual phenol, and chloroform from the elution buffer [19,20,21,22]. Furthermore, the pH and ionic strength of the RNA extraction medium, as well as RNA molecule degradation, may also affect the absorbance readings of the RNA samples [22,23]. 

The agarose gel electrophoresis of PCR assay showed positive results of dengue virus serotype 2 gene in human fluid samples of the three patients, with approximately 69 base pairs (bp) of the expected dengue virus serotype 2 amplicon bands, which can be observed in lanes P1B3, P1U3, and P1S3 of Figure S2a (see Supplementary Materials for the full-length gel), P2U3 and P2S1 of Figure S2b (see Supplementary Materials for the full-length gel), and all gel lanes of the full-length PCR products (Figure S2c, Supplementary Materials) of blood (B), urine (U), and saliva (S) samples of patient 3 (P3). Faint bands or barely discernable bands perceived in lanes P1B1, P1B2, P1U1, P1U2, P1S1, P1S2, P2B1, P2B2, P2B3, P2U1, P2U2, P2S2, and P2S3 might be attributed to a poor primer annealing process during the PCR amplification step, which led to lower quantities of PCR product being produced. Furthermore, non-specific adsorption of primers to the DNA template may also have resulted in weak positive bands being seen [24]. The low quantities of PCR product obtained may also be due to inconsistency of RNA volumes between 1.65 µL and 20.41 µL (as indicated in Table S1, Supplementary Materials) used in the cDNA synthesis step, which followed standard PCR procedure. In addition, a positive control using 500-bp gBlocks, i.e., synthetic dengue virus serotype 2 DNA, was included in the PCR electrophoretic analysis in order to verify the specificity of the PCR reaction. Additionally, we performed PCR amplification and electrophoretic analysis for DNA samples of a healthy volunteer as a negative control. No bands showing up in the gel (Figure S2d, Supplementary Materials) indicates that no dengue virus DNA was present in the healthy volunteer samples. The results from these negative reactions were confirmed using 50-bp β-actin DNAs amplified with the resulting cDNA obtained from the cDNA synthesis step of the RNA sample extracted from the healthy volunteer, and the amplicons were detected in all human biological fluid samples (the full-length gel is presented in Figure S2e, Supplementary Materials). 

Table 2 shows the statistical data obtained by the reflectance DNA biosensor and RT-PCR method in an analytical performance comparison study. The statistical paired *t*-test analysis done between the concentration of cDNA determined by the sandwich-type DNA biosensor and the RT-PCR showed *t*-values lower than the critical *t*-value of *t* = 2.776, at four degrees of freedom and 95% confidence level, for all dengue virus-infected human fluid samples; the findings suggest no significant difference between the results acquired via both methods, and the developed sandwich-type optical DNA biosensor is comparable with the standard RT-PCR reference method for dengue virus detection.

### 3.5. Comparison with Other Reported Dengue Virus DNA Biosensors

The comparison of analytical performance between the poly(*n*BA-NAS) microspheres-based sandwich-type reflectance DNA biosensor with the AuNP–PSA sphere optical label and the previously reported optical (i.e., reflectance- and fluorescence-based transducers) and electrochemical (i.e., electrochemical impedance spectroscopy, differential pulse voltammetry, and field-effect transistor) DNA biosensors for dengue virus detection is summarized in Table 3. The proposed acrylic microsphere-based optical sandwich hybridization system showed a wider detection range of 1.0 × 10^−21^ to 1.0 × 10^−12^ M, and it was ultrasensitive toward a zeptomolar-level dengue virus DNA concentration compared to most of the previously reported optical [8,25] and electrochemical [5,26,27] DNA biosensors, suggesting that the proposed DNA micro-optode holds enormous potential for the determination of low-level dengue virus DNA concentrations. This is especially advantageous in evaluation for an early warning and disaster detection system. The ultrasensitive and serotype-specific detection of dengue virus DNA could be employed as an early warning measure for detecting and predicting dengue outbreaks using mosquitoes and larvae as indicators for identifying dengue-infected zones, so as to prompt the residents to take precaution action and to facilitate the risk management of dengue outbreak by local authorities. The ability of the sandwich-type DNA micro-optode to detect dengue virus DNA in non-invasive samples, such as urine and saliva, could also reduce the risk of needle-stick injuries, bruises, and temporary local pain at the needle injection site during and after blood sample collections, which is a current bodily fluid sampling problem associated with conventional dengue virus detection methods.

The developed optical reflectance DNA biosensor shows high promise for smartphone-based image analysis with complementary metal–oxide–semiconductor/charge-coupled device (CMOS/CCD) sensors and camera systems for discrimination of color change of the DNA biosensor upon DNA sandwich hybridization detection. The novel approach of quantifying the colors of colorimetric diagnostic assays allows the high accuracy and portability of point-of-care diagnostic tests of dengue infection to be carried in resource-limited settings [28,29,30].

## 4. Conclusions

The biosensor presented herein employed a succinimide-modified acrylic microsphere-based DNA sandwich hybridization strategy, in conjunction with a gold–latex sphere optical amplification label, for ultrasensitive and serotype-specific detection of dengue virus nucleic acids. It displayed a broad range of linear response between 1.0 zM and 1.0 pM with single-base mismatch selectivity, which shows its potential as an important alternative to traditional techniques in the molecular diagnosis of viral infections, as well as for identifying bacteria or pathogenic microorganisms in human, food, and environmental samples. The analysis of actual DNA obtained from dengue virus-infected patients was shown to correspond perfectly with the standard RT-PCR method with respect to DNA quantification of dengue virus in human biological fluid samples. In addition, the use of a DNA-based biosensor for sequence-specific detection of nucleic acids can be a promising choice as a portable tool for the diagnosis of genetic diseases, drug screening, forensic analysis, and detection of emerging infectious diseases due to its low cost, facile fabrication, high sensitivity, and high specificity. As for future prospects, the nucleic acid-based biosensor will be deployed in the further development of RNA biosensing of arthropod-borne virus pathogens, eliminating the elaborate reverse transcription step, followed by cDNA amplification via PCR for direct colorimetric detection of RNA virus.

## Figures and Tables

**Table 1:** Oligonucleotides used in this study.

DNA	Base Sequences (from 5′ to 3′)
pDNA	NH_2_–(CH_2_)_6_–TTT TGT CCT GCT CTT
rDNA	CAT TTA GGC TGG GTT–(CH_2_)_3_–SH
cDNA (dengue serotype 2)	AAC CCA GCC TAA ATG AAG AGC AGG ACA AAA
1mDNA	AAC CCA TCC TAA ATG AAG AGC AGG ACA AAA
ncDNA (*Escherichia coli*)	AAC GCC GAT ACC ATT ACT TAT ACC GCG ACG

**Table 2:** Statistical comparison of two data sets on cDNA concentration determined by reflectance DNA biosensor and RT-PCR reference method by using paired *t*-test at 4 degrees of freedom and 95% confidence level.

Sample Type	Sample Code	cDNA Concentration Determined by Sandwich-Type DNA Micro-Optode (µM)	cDNA Concentration Determined by RT-PCR (µM)	*t*-Test
	P1B	7.5 ± 0.424	7.5 ± 0.109	
Blood	P2B	3.8 ± 0.937	3.4 ± 0.174	2.664
	P3B	5.3 ± 1.381	5.3 ± 0.226	
	P1U	4.9 ± 1.024	4.9 ± 0.339	
Urine	P2U	5.4 ± 1.446	5.3 ± 0.283	0.582
	P3U	2.3 ± 1.232	2.4 ± 0.073	
	P1S	4.8 ± 1.206	4.8 ± 1.659	
Saliva	P2S	3.4 ± 0.975	3.4 ± 0.248	0.407
	P3S	1.9 ± 2.101	1.9 ± 0.319	

* *t*_4_ = 2.776 (*p* = 0.05).

**Table 3:** Comparison of analytical performance between developed sandwich-type DNA micro-optode and previously reported optical and electrochemical nucleic acid biosensors in terms of dynamic linear response range, detection limit, and response time for specific detection of dengue virus.

Biosensor	Transducer	Linear Range (M)	LOD (M)	DNA Hybridization Time (min)	Reference
Succinimide-functionalized acrylic microsphere-based sandwich-type reflectance DNA biosensor with gold–latex sphere optical label	Fiber-optic reflectance spectrophotometer	1.0 × 10^−21^−1.0 × 10^−12^	1.00 × 10^−21^	90	Present work
Streptavidin-modified polyethersulfone membrane-based optical DNA biosensor with dye-entrapping liposomal nanovesicle rDNA label	Reflectometer	1.0 × 10^−9^−7.5 × 10^−7^	1.00 × 10^−9^	20	[8]
Microfluidic RNA biosensor based on PDMS microfluidic channel–glass interface	Fluorescence microscopy	1.0 × 10^−11^−2.5 × 10^−8^	1.25 × 10^−10^	20	[25]
Label-free electrochemical DNA biosensor based on platinum-coated ananoporous alumina membrane	EIS	1.0 × 10^−12^−1.0 × 10^−6^	2.70 × 10^−12^	60	[26]
Electrochemical DNA biosensor based on nanoporous alumina membrane and Fe(CN)_6_^4−^ redox label	DPV	1.0 × 10^−12^−1.0 × 10^−6^	9.55 × 10^−12^	45	[27]
Silicon nanowire-based DNA biosensor	FET	-	10.00 × 10^−15^	30	[5]

LOD = limit of detection; PDMS = poly(dimethylsiloxane); EIS = electrochemical impedance spectroscopy; DPV = differential pulse voltammetry; FET = field-effect transistor.

## Supplementary Materials

The following are available online at https://www.mdpi.com/1424-8220/20/7/1820/s1, Figure S1: The FESEM micrographs of (**a**) poly(*n*BA-NAS) and (**b**) PSA latex microspheres captured by a Zeiss Merlin field-emission scanning electron microscope operating at 15 kV; (**c**) PSA microparticles decorated with AuNPs; (**d**) the FTIR spectra of (i) poly(*n*BA-NAS) and (ii) PSA copolymer microspheres acquired by Agilent Cary 630 FTIR spectrometer from 500–4000 cm^−1^; (**e**) the UV–Vis absorption spectrum and (**f**) TEM image of colloid AuNPs synthesized by citrate reduction method; Figure S2: PCR electrophoretic results for dengue virus serotype 2 detection in blood, urine, and saliva samples collected from patient 1 (**a**), patient 2 (**b**), patient 3 (**c**), and a healthy volunteer (**d**,**e**). The lane numbers indicate patients’ sample codes. A positive control of amplification was included, denoted as “C”. Amplicon size: 69 bp (dengue virus serotype 2) and 50 bp (β-actin). DNA ladder used: O’Range Ruler 20 bp. PCR products were detected on 4% agarose in TBE buffer at 90 V for 60 min; Table S1: The RNA concentrations extracted from blood (B), urine (U), and saliva (S) samples of patient 1 (P1), patient 2 (P2), patient 3 (P3), and a healthy volunteer (H) based on A260/280 and A260/230 absorbance ratios by using a NanoDrop spectrophotometer. The right column shows the volume of RNA used in the cDNA synthesis step.

## References

[1] Darwish T.N., Alias Y., Khor S.M. (2015). An introduction to dengue-disease diagnostics. Trends Anal. Chem..

[2] (2014). World Health Organization, Global Health Estimates 2014 Summary Tables: Deaths by Cause, Age and sex, by World Bank Income Group Category. 2000–2012. http://www.who.int/healthinfo/global_burden_disease/en/.

[3] Parkash Q., Shueb R.H. (2015). Diagnosis of dengue infection using conventional and biosensor based techniques. Viruses.

[4] Cheng M.S., Ho J.S., Tan C.H., Wong J.P., Ng L.C., Toh C.S. (2012). Development of an electrochemical membrane-based nanobiosenor for ultrasensitive detection of dengue virus. Anal. Chim. Acta.

[5] Zhang G., Zhang L., Huang M.J., Luo Z.H.H., Tay G.K.I., Lim E.A., Kang T.G., Chen Y. (2010). Silicon nanowire biosensor for highly sensitive and rapid detection of dengue virus. Sens. Actuators B Chem..

[6] Nascimento H.P.O., Oliveira M.D.L., Melo C.P., Silva G.J.L., Cordeiro M.T., Andrade C.A.S. (2011). An impedimetric biosensor for detection of dengue serotype at picomolar concentration based on gold nanoparticles-polyaniline hybrid composites. Colloids Surf. B.

[7] Fletcher S.J., Philips L.W., Milligan A.S., Rodda S.J. (2010). Toward specific detection of dengue virus serotypes using a novel modular biosensor. Biosens. Bioelectron..

[8] Baeumner A.J., Schlesinger N.A., Slutzki N.S., Romano J., Lee E.M., Montagna R.A. (2002). Biosensor for dengue virus detection: Sensitive, rapid, and serotype specific. Anal. Chem..

[9] Ulianas A., Lee Y.H., Sharina A.H., Tan L.L. (2012). An electrochemical DNA microbiosensor based on succinimide-modified acrylic microspheres. Sensors.

[10] Liang Z., Zhang L., Wang L., Song S., Fan C., Li G. (2007). A centrifugation-based method for preparation of gold nanoparticles and its application in biodetection. Int. J. Mol. Sci..

[11] Kuan G.C., Sheng L.P., Rijiravanich P., Marimuthu K., Ravichandran M., Yin L.S., Lertanantawong B., Surareungchai W. (2013). Gold-nanoparticle based electrochemical DNA sensor for the detection of fish pathogen Aphanomyces invadans. Talanta.

[12] Mizuno Y., Kotaki A., Harada F., Tajima S., Kurane I., Takasaki T. (2007). Confirmation of dengue virus infection by detection of dengue virus type 1 genome in urine and saliva but not in plasma. Trans. R. Soc. Trop. Med. Hyg..

[13] Ulianas A., Lee Y.H., Musa A. (2011). A biosensor for urea from succinimide-modified acrylic microspheres based on reflectance transduction. Sensors.

[14] Bastús N.G., Comenge J., Puntes V. (2011). Kinetically controlled seeded growth synthesis of citrate-stabilized gold nanoparticles of up to 200 nm: Size focusing versus ostwald ripening. Langmuir.

[15] Hames B.B., Higgins S.J. (1985). Nucleic Acid Hybridisation—A Practical Approach.

[16] Metzenberg S. (2007). Working with DNA: The Basics.

[17] Chan C.P., Choi J.W., Cao K.Y., Wang M., Gao Y., Zhuo D.H., Di B., Xu H.F., Leung M.F., Bergmann A. (2006). Detection of serum neopterin for early assessment of dengue virus infection. J. Infect..

[18] Park J.S., Na H., Min D., Kim D. (2013). Desorption of single-stranded nucleic acids from graphene oxide by disruption of hydrogen bonding. Analyst.

[19] Simonian M.H., Smith J.A. (2006). Spectrophotometric and colorimetric determination of protein concentration. Curr. Protoc. Mol. Biol..

[20] Stollar V., Schlesinger R.W., Stevens T.M. (1967). Studies on the nature of dengue viruses: III. RNA synthesis in cells infected with type 2 dengue virus. Virology.

[21] Ausubel F.M., Brent R., Kingston R.E., Moore D.D., Seidman J.G., Smith J.A., Struhl K. (1999). Current Protocols in Molecular Biology.

[22] Wilfinger W., Mackey K., Chomczynski P. (1997). Effect of pH and ionic strength on the spectrophotometric assessment of nucleic acid purity. Biotechniques.

[23] Li Z., Reimers S., Pandit S., Deutscher M.P. (2002). RNA quality control: Degradation of defective transfer RNA. EMBO J..

[24] Radonic A., Thulke S., Mackay I.M., Landt O., Siegert W., Nitsche A. (2004). Guideline to reference gene selection for quantitative real-time PCR. Biochem. Biophys. Res. Commun..

[25] Zaytseva N.V., Montagna R.A., Baeumner A.J. (2005). Microfluidic biosensor for the serotype-specific detection of dengue virus RNA. Anal. Chem..

[26] Deng J., Toh C.S. (2013). Impedimetric DNA biosensor based on a nanoporous alumina membrane for the detection of the specific oligonucleotide sequence of dengue virus. Sensors.

[27] Rai V.C., Hapuarachchi H.C., Ng L.C., Soh S.H., Leo Y.S., Toh C.S. (2012). Ultrasensitive cDNA detection of dengue virus rna using electrochemical nanoporous membrane-based biosensor. PLoS ONE.

[28] Priye A., Ball C.S., Meagher R.J. (2018). Colorimetric-luminance readout for quantitative analysis of fluorescence signals with a smartphone CMOS sensor. Anal. Chem..

[29] Shen L., Hagen J.A., Papautsky I. (2012). Point-of-care colorimetric detection with a smartphone. Lab Chip.

[30] Priye A., Bird S.W., Light Y.K., Ball C.S., Negrete O.A., Meagher R.J. (2017). A smartphone-based diagnostic platform for rapid detection of Zika, chikungunya, and dengue viruses. Sci. Rep..

