# Supplementary material for: Sandwich-Type DNA Micro-Optode Based on Gold–Latex Spheres Label for Reflectance Dengue Virus Detection
Source: Sensors (Basel). 2020 Mar 25;20(7):1820. doi: 10.3390/s20071820 (PMC7180460; doi:10.3390/s20071820)
Supplement: Supplementary file 1 [file sensors-20-01820-s001.pdf]

Supplementary Material

Figure 1. The FESEM micrographs of (**a**) poly(nBA-NAS) and (**b**) PSA latex microspheres captured by Zeiss Merlin field-emission scanning electron microscope operating at 15 kV; (**c**) PSA microparticles decorated with AuNPs; (**d**) the FTIR spectra of i) poly(*n*BA-NAS) and ii) PSA copolymer microspheres acquired by Agilent Cary 630 FTIR spectrometer from 500-4000 cm^{-1}; (**e**) the UV-Vis absorption spectrum and (**f**) TEM image of colloid AuNPs synthesized by citrate reduction method.

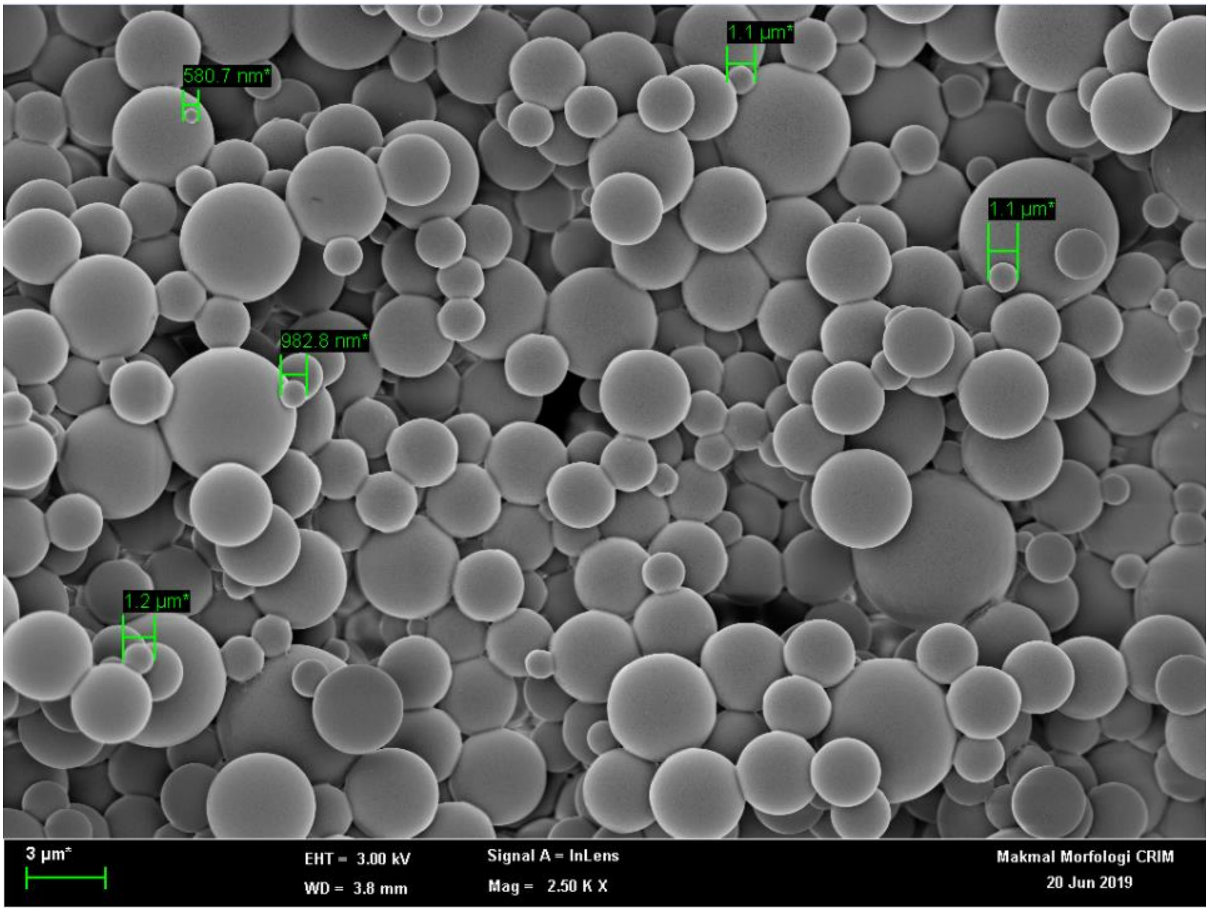

(**a**)

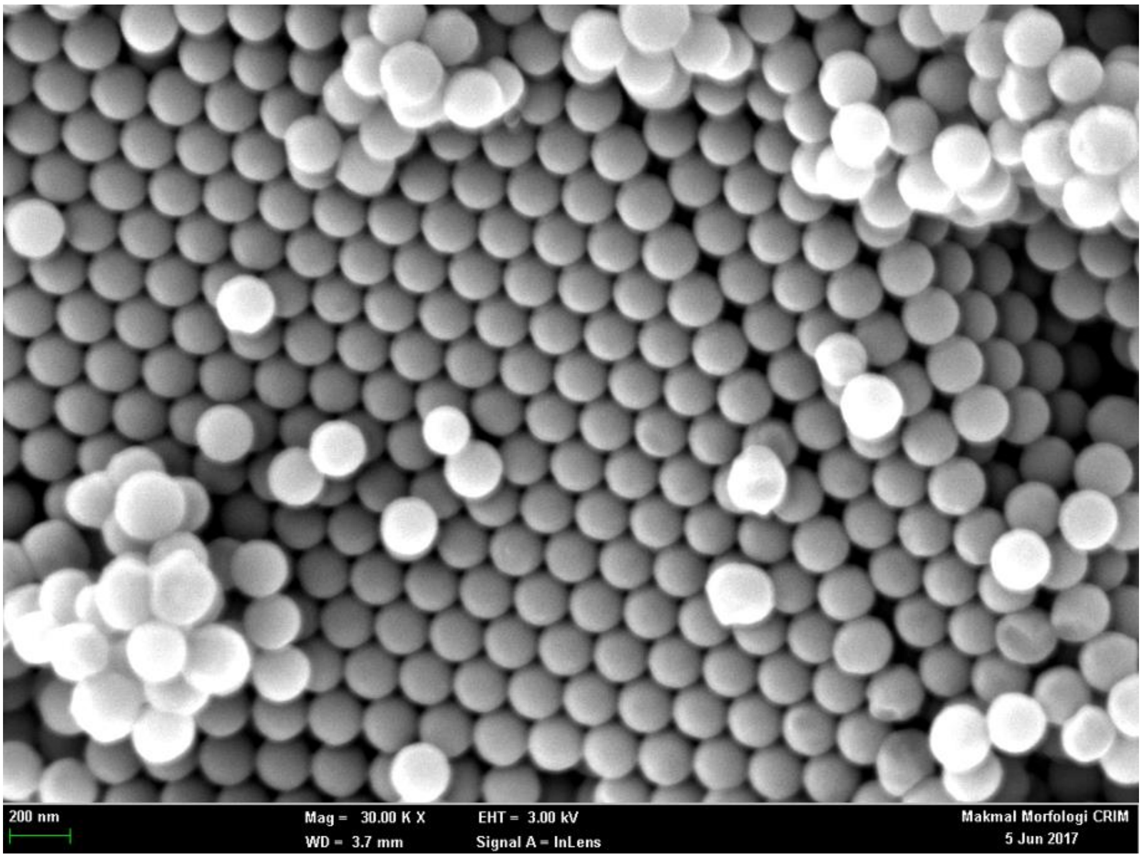
200 nm
Mag = 30.00 K X
WD = 3.7 mm
EHT = 3.00 kV
Signal A = InLens
Makmal Morfologi CRIM
5 Jun 2017
(**b**)

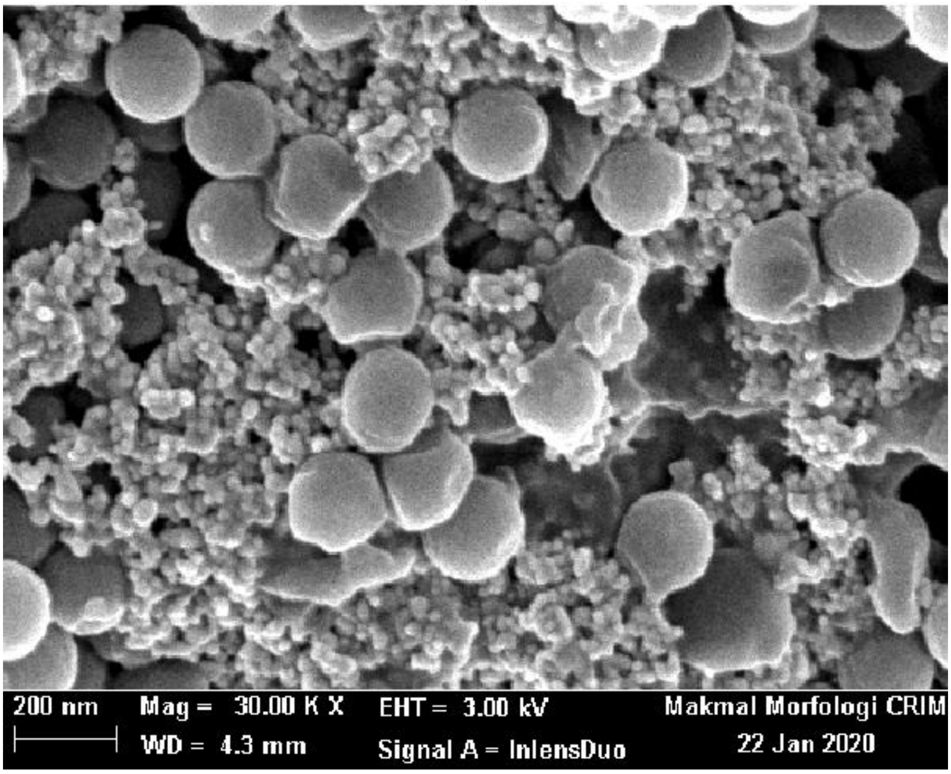
200 nm
Mag = 30.00 K X
WD = 4.3 mm
EHT = 3.00 kV
Signal A = InlensDuo
Makmal Morfologi CRIM
22 Jan 2020
(**c**)

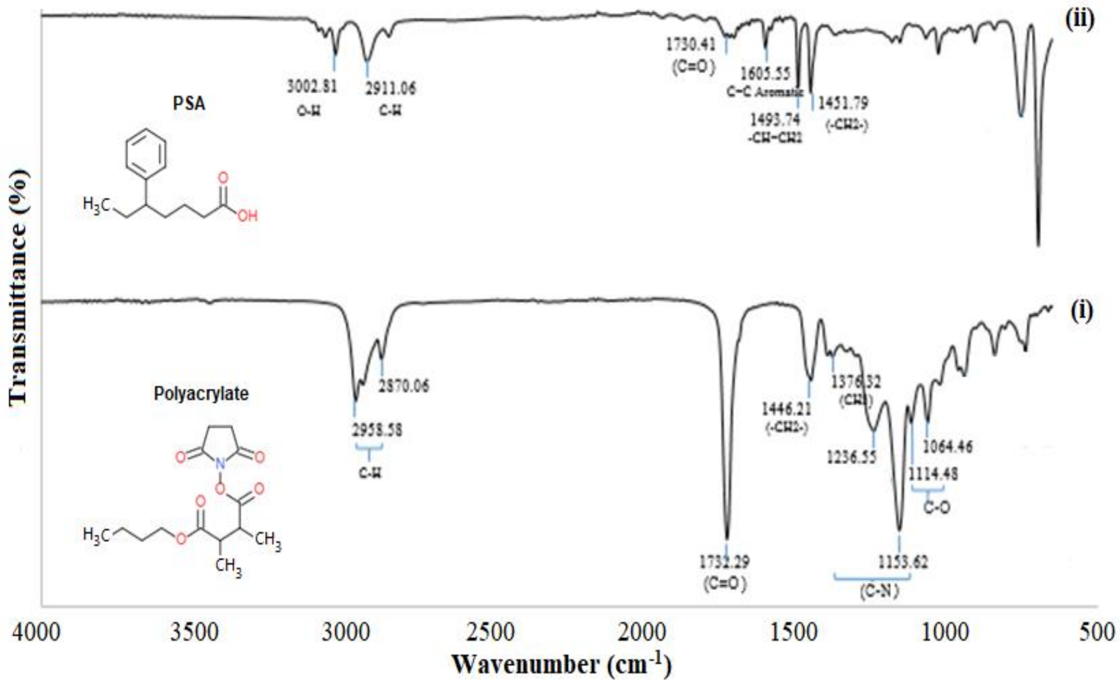
(ii)
(i)
PSA
Polyacrylate
3002.81
O-H
2911.06
C-H
1730.41
(C=O)
1605.55
C-C Aromatic
1493.74
-CH-CH1
1451.79
(-CH2-)
2870.06
2958.58
C-H
1732.29
(C=O)
1446.21
(-CH2-)
1376.32
(CH)
1236.55
1064.46
1114.48
C-O
1153.62
(C-N)
Transmittance (%)
4000
3500
3000
2500
2000
1500
1000
500
Wavenumber (cm-1)

(d)

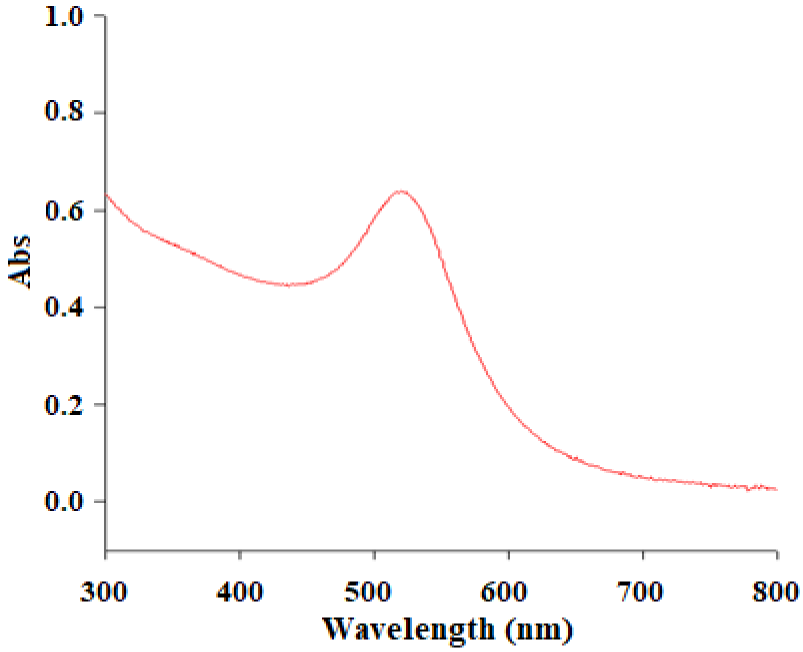
1.0
0.8
0.6
0.4
0.2
0.0
Abs
300
400
500
600
700
800
Wavelength (nm)

(e)

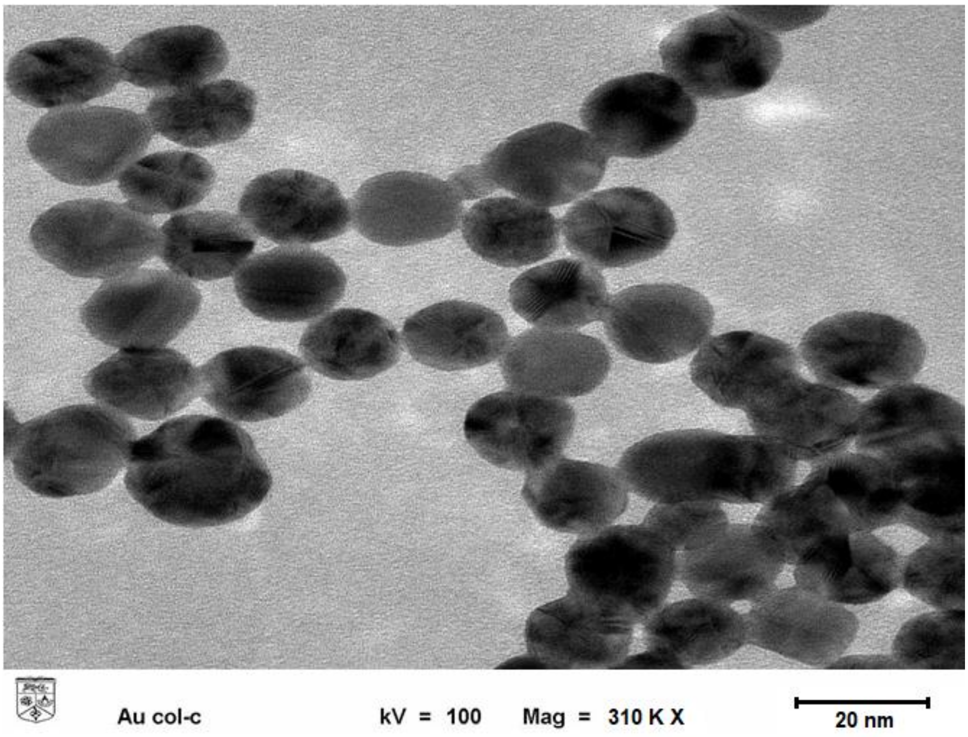

(**f**)

Figure S2 PCR electrophoretic results for dengue virus serotype 2 detection in blood, urine and saliva samples collected from patient 1 (**a**), patient 2 (**b**), patient 3 (**c**) and healthy volunteer (d and e). The lane numbers indicate patients' sample codes. Positive control of amplification was included and denoted as 'C'. Amplicon size: 69 bp (dengue virus serotype 2) and 50 bp (β-actin). DNA ladder used: O'Range Ruler 20 bp. PCR products were detected on 4% Agarose in TBE buffer at 90 V for 60 min.

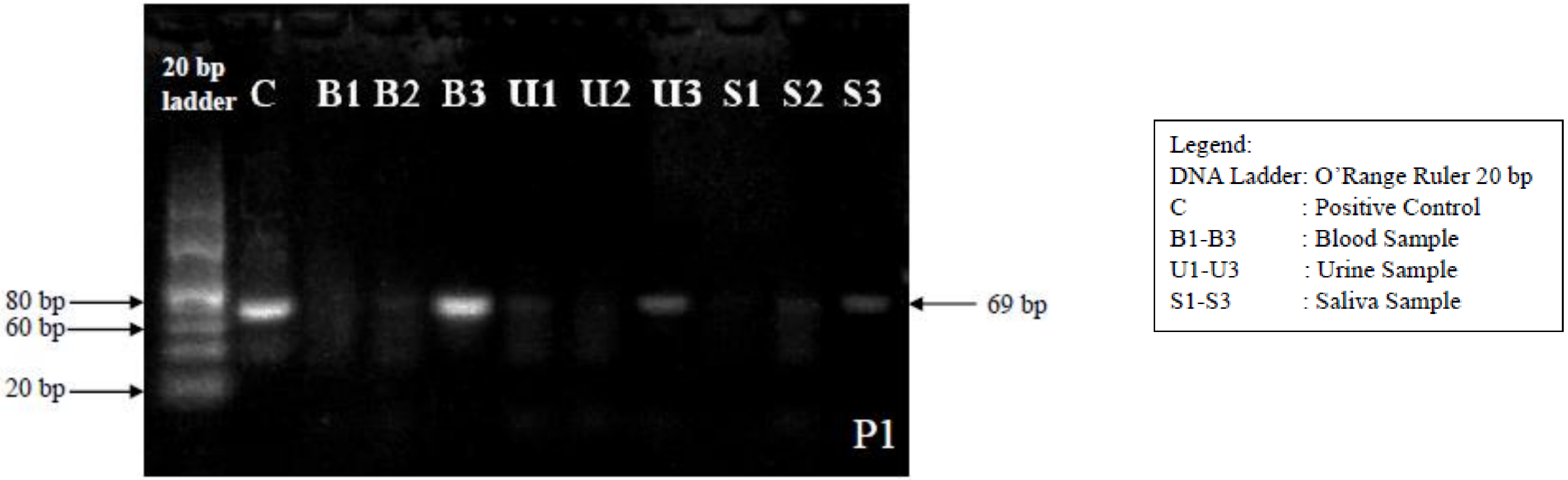

(**a**)

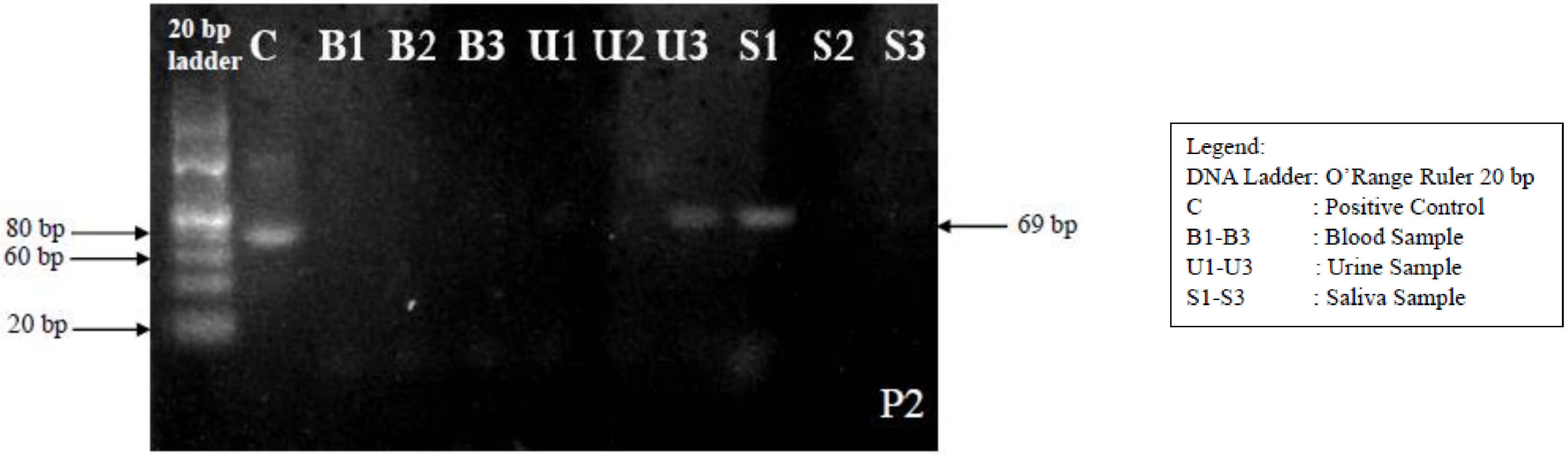
20 bp ladder
C
B1
B2
B3
U1
U2
U3
S1
S2
S3
80 bp
60 bp
20 bp
69 bp
P2
Legend:
DNA Ladder: O'Range Ruler 20 bp
C : Positive Control
B1-B3 : Blood Sample
U1-U3 : Urine Sample
S1-S3 : Saliva Sample

(**b**)

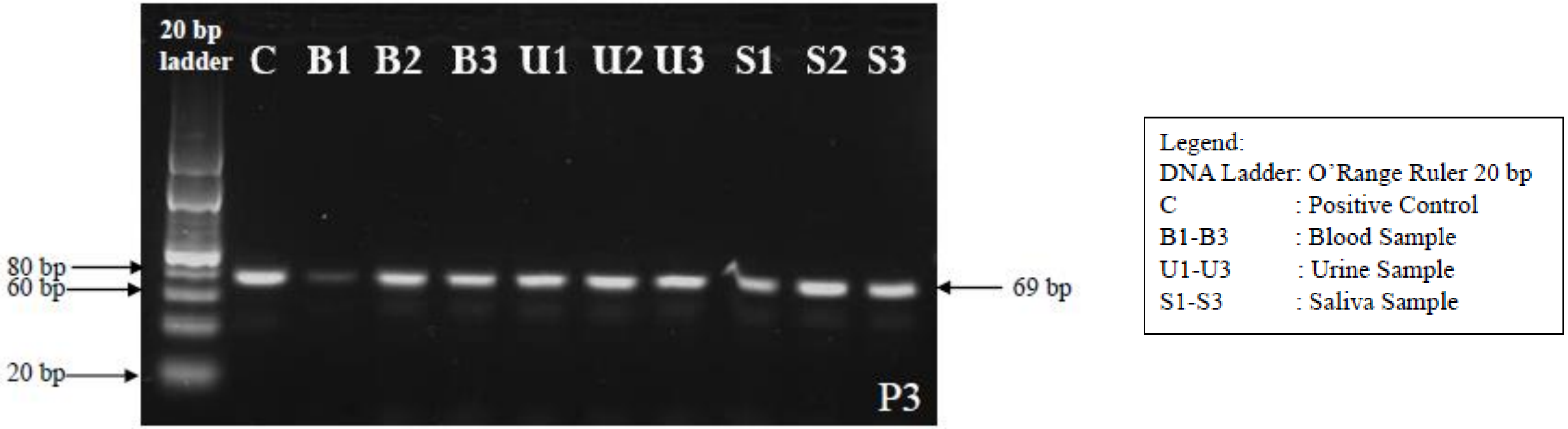
20 bp ladder
C
B1
B2
B3
U1
U2
U3
S1
S2
S3
80 bp
60 bp
20 bp
69 bp
P3
Legend:
DNA Ladder: O'Range Ruler 20 bp
C : Positive Control
B1-B3 : Blood Sample
U1-U3 : Urine Sample
S1-S3 : Saliva Sample

(**c**)

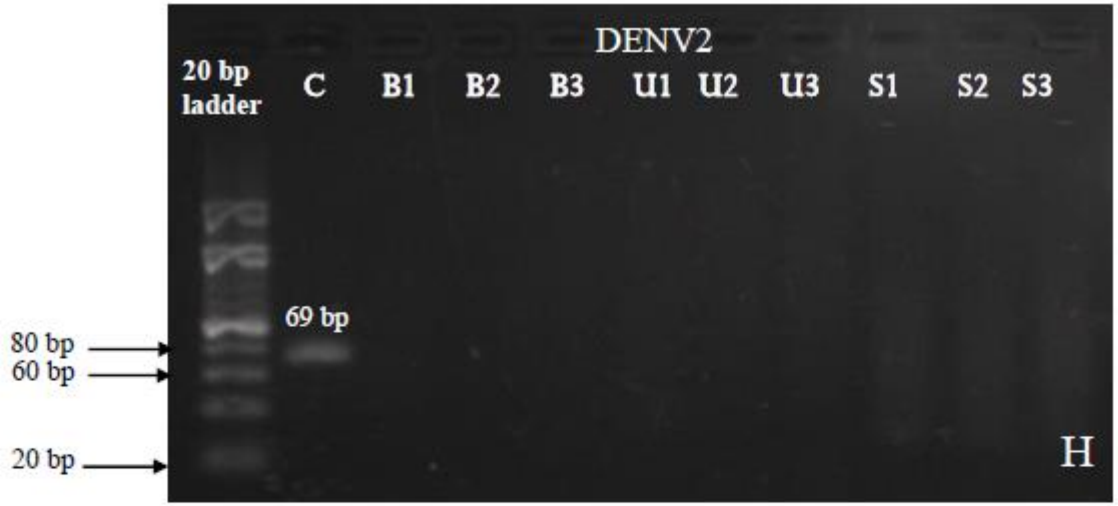

Legend:
DNA Ladder: O'Range Ruler 20 bp
C : Positive Control
B1-B3 : Blood Sample
U1-U3 : Urine Sample
S1-S3 : Saliva Sample

(**d**)

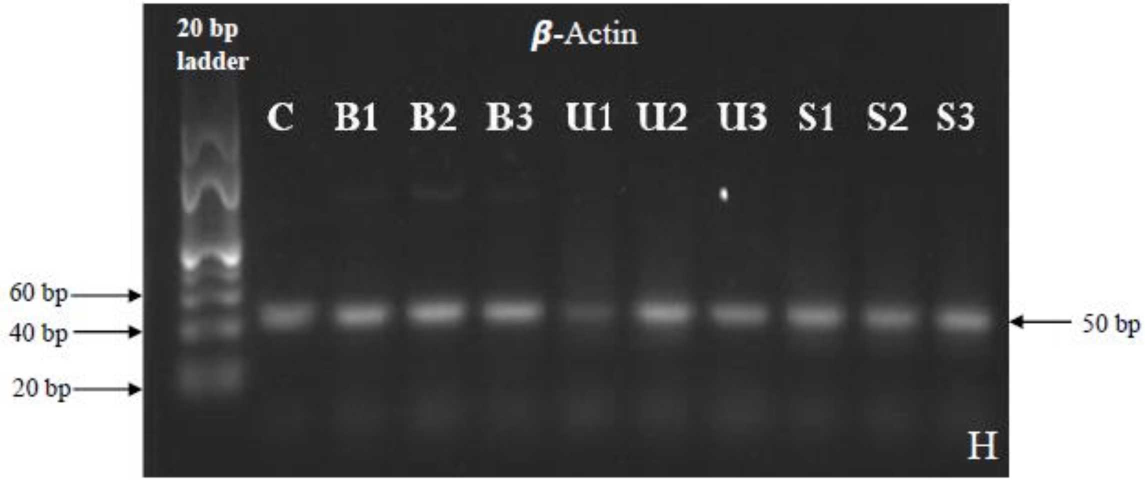

Legend:
DNA Ladder: O'Range Ruler 20 bp
C : Positive Control
B1-B3 : Blood Sample
U1-U3 : Urine Sample
S1-S3 : Saliva Sample

(**e**)

Table 1. The RNA concentrations extracted from blood (B), urine (U) and saliva (S) samples of patient 1 (P1), patient 2 (P2), patient 3 (P3) and healthy voluteer (H) based on A260/280 and A260/230 absorbance ratios by using NanoDrop spectrophotometer. The right column shows the volume of RNA used in cDNA synthesis step. The results are presented as mean (n = 3).

Sample ID	RNA concentration (ng/μL)	A260/280	A260/230	Volume for 100 ng RNA (μL)
P1B1	43.6	1.38	0.17	2.29
P1B2	22.6	1.70	0.06	4.42
P1B3	39.0	1.56	0.30	2.56
P1U1	44.4	1.49	0.30	2.25
P1U2	49.8	1.58	0.37	2.01
P1U3	60.6	1.46	0.46	1.65
P1S1	40.8	1.62	0.20	2.45
P1S2	40.7	1.51	0.30	2.46
P1S3	41.8	1.73	0.29	2.39
P2B1	5.5	1.56	0.04	18.18
P2B2	6.0	1.55	0.05	16.67
P2B3	5.7	1.78	0.04	17.54
P2U1	5.2	1.72	0.04	19.23
P2U2	5.9	1.38	0.03	16.95
P2U3	5.5	1.85	0.04	18.18
P2S1	6.0	1.74	0.05	16.67
P2S2	5.9	1.11	0.03	16.95
P2S3	4.9	1.21	0.04	20.41
P3B1	5.5	1.56	0.04	18.18
P3B2	5.7	1.55	0.03	17.54
P3B3	5.5	1.68	0.03	18.18
P3U1	5.9	2.01	0.05	16.95
P3U2	4.9	1.11	0.04	20.41
P3U3	5.2	1.68	0.03	19.23
P3S1	6.1	1.88	0.03	16.39
P3S2	5.6	2.09	0.04	17.86
P3S3	6.2	1.89	0.04	16.13
HB1	10.0	2.57	0.03	10.00
HB2	6.4	1.17	0.02	15.63
HB3	10.3	1.87	0.03	9.71
HU1	48.8	1.68	0.27	2.05
HU2	43.0	2.60	0.31	2.33
HU3	12.8	1.69	0.09	7.81
HS1	33.2	1.66	0.25	3.01
HS2	40.1	1.78	0.23	2.49
HS3	47.7	1.74	0.19	2.10
